# Ambient intelligence for long-term diabetes care (AmILCare). Qualitative analysis of patients’ expectations and attitudes toward interactive technology

**DOI:** 10.1007/s12020-021-02694-1

**Published:** 2021-03-25

**Authors:** Marina Trento, Marta Franceschini, Paolo Fornengo, Lucia Tricarico, Aurora Mazzeo, Stefania Bertello, Alessandra Clerico, Salvatore Oleandri, Mario Chiesa, Anna Di Leva, Lorena Charrier, Franco Cavallo, Massimo Porta

**Affiliations:** 1 Laboratory of Clinical Pedagogy, Department of Medical Sciences, University of Turin, Turin, Italy; 2 Diabetes Unit, Azienda Sanitaria Città di Torino, Turin, Italy; 3 Links Foundation, Polytechnic University of Turin, Turin, Italy; 4 Department of Public Health and Paediatric Sciences, University of Turin, Turin, Italy

Ambient intelligence (AmI) refers to environments in which electronic devices work in concert to support people in their everyday [1, 2] life activities, tasks, and rituals [3] in an intuitive way. AmILCare (Ambient Intelligence for Long-term diabetes Care) is a project aimed at developing Information and Communication Technology solutions, based upon an AmI paradigm and co-designed with patients, to support healthy lifestyles in people with non insulin-treated Type 2 Diabetes T2D [4]. Based upon an integrated evaluation of the data collected by wearable sensors, smart objects will communicate to patients whether their daily activities have been more or less conducive to maintaining a satisfactory metabolic control and preventing complications. Feedbacks will take the form of emotional messages (light, sound or cinematics effects, text/vocal messages) from networked wearable devices or home appliances [5].

In the first phase of AmILCare, we aimed at probing the meaning that people with non-insulin-treated T2D attribute to technology for self-care and their willingness to participate in co-designing AmI solutions.

## Research design and methods

Thirty-four patients with non-insulin-treated T2D were recruited consecutively during their outpatient visits. Inclusion criteria were age <80, at least 1-year previous attendance in our clinic and treatment by lifestyle alone or with non-insulin anti-hyperglycaemic agents. Recruitment began in September 2019 and forcibly stopped at the end of January 2020, due to the COVID-19 pandemic.

The study was in accordance with the 2013 Helsinki Declaration and approved by the Institutional Ethics Committees of Città della Salute e della Scienza di Torino and Ordine Mauriziano di Torino. All patients signed an informed consent to participate.

### Socio-demographic and clinical variables

Socio-demographic and clinical variables obtained within the previous 12 months are listed in Table 1. LDL Cholesterol and eGFR were calculated according to Friedewald et al. [6] and Cockroft and Gault formula [7], respectively. Blood pressure was measured after 5 min lying using a mercury sphygmomanometer. Fundus examination was by digital retinal photography, graded according to Italian guidelines [8, 9]. None of the patients suffered from clinically evident coronary, peripheral, or cerebral vascular disease.

**Table 1:** Socio-demographic and clinical variables of the patients interviewed

Sex	*M* = 19; *F* = 15
Age	72 (63–73.50)
Schooling (primary school/middle school/high school/university degree)	5/17/9/3
Occupation (retired/active in work)	13/21
Social status (living alone/married)	6/28
Smoking (never/currently/stopped)	13/6/15
Family history of DM (no/yes)	7/27
Known duration of diabetes (years)	16.50 (11.75–20)
Anti-hyperglycemic treatment (lifestyle only/anti-hyperglycemic agents)	3/31
Anti-hyperthensive treatment (no/yes)	5/29
Group Care (no/yes)	23/11
Body weight (kg)	72 (65.5–84.5)
Body Mass Index (kg/m^2^)	27.5 (24.75–30)
Fasting blood glucose (mg/dl)	136.5 (126.25–159.5)
HbA_1c_ (percent of total Hb)	7.05 (6.5–7.725)
HbA1_c_ (mmol/mol)	51.5 (48–61)
Systolic blood pressure (mmHg)	138.5 (130–140.5)
Diastolic blood pressure (mmHg)	80 (70–80)
Total cholesterol (mg/dl)	160.5 (140.5–179.5)
HDL cholesterol (mg/dl)	41.5 (36–59)
LDL cholesterol (mg/dl)	82.9 (68.7–106.4)
Triglyceride (mg/dl)	116.5 (94–150)
Creatinine (mg/dl)	0.775 (0.6375–0.9875)
ACR	0.95 (0.2375–2.03)
eGFR (ml/min)	79.65 (67.975–100.05)
Diabetic retinopathy (absent/mild/more severe)	25/7/2
Foot lesions (none)	34

Absolute frequencies are used for categorical variables and medians and IQ range for continuous variables

### Interview protocol and psychometric evaluation

The patients were administered a 50 min structured interview, adapted from a tool to analyze the usability of technology by adults [10]. The dimensions explored and the questions are listed in Table 2. Interviews were done by two researchers and transcribed verbatim. Analysis of the interviews included objective and quantitative descriptions of the concepts expressed by the patients.

**Table 2:** Dimensions, questions and answers to the protocol interview

	*N* (%)
**(a) Interest in technology:** “*There is a growing interest in technologies that can be used to help people support healthy lives. What do you think?*”	
* Not interested*	3 (8.8)
* Interested*	31 (91.2)
**(b) Use and interest in technology in connection to personal health:** “*Do you use technologies to stay healthy?*”	
* No use*	9 (26.5)
* Use for blood sugar control*	1 (2.9)
* Use for blood pressure control*	15 (44.1)
* Use for both*	9 (26.5)
**(c) Personal motivation:** “*Why do you use these technologies?”*	
* Don’t know*	2 (5.9)
* Personal safety*	6 (17.6)
* To check health*	22 (64.7)
* Both previous items*	4 (11.8)
**(d) Preferences for sharing health information:** “*Whom would you prefer to share the information with?”*	
* None*	3 (8.8)
* Physician*	19 (55.9)
* Family*	7 (20.6)
* Both previous items*	5 (14.7)
**(e) Support and Help:** “*What would you like to be included in these devices to help manage your diabetes?”*	
* None*	5 (14.7)
* To help control nutrition*	21 (61.8)
* To help control diabetes*	3 (8.8)
* To avoid pain*	5 (14.7)
**(f) Design:** “*What should the features of the device be?”*	
* None*	6 (17.6)
* Small and discreet object*	16 (47.1)
* Nonintrusive object*	7 (20.6)
* Both previous items*	5 (14.7)
**(g) Design:** “*Would you like to design the device?”*	
* No*	27 (79.4)
* Yes*	7 (20.6)
**(h) Intention to participate in co-designing technology:** “*Would you be interested in interacting with those who design technology to guide the design process of products specifically geared toward people with type 2 diabetes?”*	
* No*	11 (32.4)
* Yes*	23 (67.6)

Quality of Life was measured using a 39-item DQoL/Mod version adapted for patients with T2D, translated, and revalidated into Italian [11]. The dimensions measured are: the 14-item Satisfaction (14 items), impact of diabetes (20 items), and diabetes-related anxiety (5 items). Answers are along 5-point Likert scales, from 1 (very satisfied) to 5 (very dissatisfied). Scores range from 39 (best quality of life) to 195 (worst quality).

Self-esteem was measured by the Rosenberg Scale [12], including ten items to be answered along 4-point Likert scales. Scores ranges from 10 to 40, higher values corresponding to better self-esteem.

### Statistical methods

Data are shown as absolute and relative frequencies for categorical variables and as median and interquartile range for continuous variables. Distribution of answers to the protocol interview were compared by gender by means of a chi-square test.

## Results

Although we had planned to recruit 50 subjects, the COVID-19 pandemic forced us to stop at 34 interviews. The patients had a median age of 72 years and 16.5 years disease duration (Table 1).

DQoL Total (*M* = 64.5—IQ: 60.75–73.75), and its dimensions Satisfaction (*M* = 29—IQ: 26–32), impact (*M* = 27.5—IQ: 24–33.25), and worry (*M* = 8—IQ: 6–10.25) suggested good quality of life, similarly to self-esteem scores (*M* = 34.5—IQ: 30–37.25).

Table 2 shows the distribution of answers to the interviews. Nearly all persons showed interest in the AmI project, and technology had a positive connotation to them. Personal motivation to use technology included checking health, personal safety, or both. Except for three patients, most wished to share personal health information with their physician or family.

There were no gender differences in the propensity to use technology for health control, personal motivation, whom to share health information with or support in disease care. Regarding the characteristics of technology aids, women favored small discreet objects, men valued non-intrusiveness (*p* = 0.05). Women were more inclined to draft an object for the AmILcare project (*p* = 0.10).

Patients aiming to monitor their health and wishing to share information with family showed trends to better scores on the quality of life scale for complications; lower HbA1c scores were recorded among those interested in the construction of new devices.

## Discussion

Integrating technology into health care shifts accountability from professionals to patients, redefining their role from passive recipients to active participants and requiring that they acquire specific competences [13], a main problem for non-technologically literate people. AmI aims at providing nonintrusive, intuitive solutions to make technology easily available to people. A key point of AmILCare is the involvement of patients with diabetes in co-designing smart objects that will support them in their daily choices to maintain good control [14]. This study aimed at exploring how people with T2D perceive technology, whether they use it in daily life, and their willingness to participate in developing solutions they might benefit from. Since self-management involves daily choices made without the support of operators, people with T2D need support that is simple and easy to use. AmILCare aims at developing smart objects that will collect and process data about patients’ behaviors and clinical variables and provide them with feedbacks to improve control.

The interviews revealed the willingness of patients to participate in the project and some of their preferences. As shown in Table 2, they wish to control blood pressure, presumably because they experience this noninvasive manouvre during medical examinations [15], and prefer to discuss their health problems with their doctor, as in our clinic they spend more time talking with physicians than other professionals [16]. In terms of benefits expected of an AmI environment, women preferred to receive advice on nutrition but did not ask for specific information. As eating behaviors may change over time, choices need to be adapted accordingly [17].

The request for small, nonintrusive objects is in line with the AmI paradigm [18], stating that technology should be supportive and respect expectations without becoming intrusive. Co-production of technology is a complex process, empowering and at the same time exploiting actors. It needs theoretical tools [18, 19] as a user’s perception is affected by a continuum from expectations to experience with a given service [19]. Our interviews showed a propensity by women to get involved in co-design, a useful aspect to nurture support in managing the disease [20]. Opportunities for training and involvement enable patients to personally acknowledge the challenges inherent in treating diabetes, a perspective that helps establish them as partners in decision-making [20]. This is, to our knowledge, the first study to inquire directly about the attitudes of people with non insulin-treated T2D towards health care technology, with a specific insight into AmI solutions. A limitation is the small number of patients. Unfortunately, the outbreak of the Covid-19 pandemic made it unsafe to continue summoning patients for interviews in presence. However, the interviews helped reveal interests and expectations and gave indications about the characteristics that smart objects should have to make them acceptable and usable. This approach could be extended to other interventions aimed at promoting patient participation and wish to collaborate in decision-making and disease self-management.
